# Palbociclib Capsule: A Bioequivalence Study in Healthy Subjects Under Fed Conditions to Compare Two Formulations

**DOI:** 10.3390/pharmaceutics18020175

**Published:** 2026-01-29

**Authors:** Marcelo Gomes Davanço, Thaís Pereira Vespasiano, Jessé Moisan, Maira Eduarda Zanin, Gilberto Carlos Ruggiero Bernasconi, Marcia Aparecida Antonio, Oscar Gonzalez, Milesa Sarmiento, Mélanie Groleau

**Affiliations:** 1 Medical & Clinical Affairs Department, United Medical Ltda. (a Knight Therapeutics Company), Sao Paulo 04085-001, SP, Brazil; thais.vespasiano@knighttx.com (T.P.V.); 2 Medical & Clinical Affairs Department, Knight Therapeutics Inc., Montreal, QC H4M 2P2, Canada; jmoisan@knighttx.com (J.M.); mgroleau@knighttx.com (M.G.); 3 Unidade Integrada de Farmacologia e Gastroenterologia (UNIFAG), Universidade São Francisco, Bragança Paulista 12916-900, SP, Brazil; maira.zanin@unifag.com.br (M.E.Z.); gilberto.bernasconi@unifag.com.br (G.C.R.B.); marcia.antonio@unifag.com.br (M.A.A.); 4 R&D Department, Laboratorio LKM S.A. (a Knight Therapeutics Company), Buenos Aires C1179, Argentina; oscar.gonzalez@knighttx.com (O.G.); milesa.sarmiento@knighttx.com (M.S.)

**Keywords:** palbociclib, bioequivalence, pharmacokinetics, bioavailability, generics, advanced breast cancer

## Abstract

**Background:** Globally, breast cancer is the most frequently diagnosed neoplasm among women, with an estimated 2.3 million new cases reported in 2022. Treatment for hormone receptor-positive (HR+) advanced breast cancer includes aromatase inhibitors and CDK4/6 inhibitors such as palbociclib. **Objective:** This study evaluated the bioequivalence and tolerability of two palbociclib capsule formulations to support the regulatory approval of a branded generic product in Latin America countries. **Methods:** Healthy participants were enrolled in an open-label, randomized, single-dose study using a two-treatment, two-sequence, two-period crossover design. Study participants received a single-dose test product, a palbociclib 125 mg capsule (Laboratório LKM S.A., Argentina), and a reference product, an Ibrance^®^ 125 mg capsule (Pfizer Manufacturing Deutschland GmbH), under fed conditions separated by a 14-day washout period. Blood samples were obtained at scheduled intervals over a 72 h period following administration, and the palbociclib plasma concentrations were determined using a validated LC-MS/MS method. Pharmacokinetic parameters were computed via non-compartmental analysis methods. A total of 52 healthy subjects were enrolled, and 50 subjects completed the study. **Results:** The geometric mean ratios (90% confidence intervals) for C_max_ and AUC_0–72_ were 107.07% (101.98–112.42) and 109.77% (106.51–113.13), respectively. **Conclusions:** Both formulations were well-tolerated in healthy subjects. In accordance with regulatory standards, bioequivalence between the test formulation and the reference product was successfully demonstrated.

## 1. Introduction

Breast cancer is the most commonly diagnosed malignancy in women globally and represents the second most prevalent cancer in the overall population. In 2022, it was estimated that approximately 2.3 million new cases of breast cancer were diagnosed globally, resulting in around 670,000 related deaths [1]. Breast cancer is the leading cause of cancer-related deaths among women in Latin America, with incidence rates varying according to socioeconomic factors, access to healthcare services, and screening programs [2]. Mortality remains high due to late-stage diagnoses and barriers to treatment access.

Breast cancer is a complex disease, with many subtypes characterized by different histological and molecular markers, and its identification has aided in the prognosis of new cases [3]. The main markers are the hormonal receptors, such as the estrogen receptor (ER), the progesterone receptor (PR), and the epidermal growth factor receptor 2 (HER2) [3]. In this context, selective CDK4/6 inhibitors are promising therapies for various cancers, particularly HR+ breast cancer [3,4,5,6].

Palbociclib, a selective CDK4/6 inhibitor of the second generation, was approved by the U.S. Food and Drug Administration (FDA) in February 2015 for use in postmenopausal women with ER-positive, HER2-negative advanced breast cancer [6,7,8]. Palbociclib acts reversibly by inhibiting CDK4/6 enzymes, preventing their binding to cyclin D1 and thus blocking the phosphorylation of the Rb protein. In this way, it prevents the progression of cellular division in the G1 phase, inhibiting tumor progression [3,6,8].

Palbociclib is available in capsule and film-coated tablet formulations, each in 75 mg, 100 mg, and 125 mg strengths. Capsules require fed administration, as food reduces inter-individual pharmacokinetic variability. Based on the Ibrance^®^ leaflet [9], the time to reach maximum concentration (t_max_) for palbociclib is generally between 6 and 12 h after oral administration and the elimination half-life is approximately 28 h. The mean absolute bioavailability of palbociclib following a single oral dose of 125 mg is approximately 46%. Within the dosage range of 25 mg to 225 mg, both AUC and C_max_ increase in an approximately dose-proportional manner. Steady state is achieved in about 8 days after repeated once-daily administration [9].

Generic drug products play a crucial role in the context of oncology treatments, as they reduce costs and increase the availability of essential therapies, such as palbociclib, particularly in resource-limited health systems of Latin America countries. For the registration of generic palbociclib capsule products, the FDA and the European Medicines Agency (EMA) recommend conducting a single-dose bioequivalence study in healthy subjects in a fed state, which is aligned with the administration mode described for the approved labeling for palbociclib capsules. This recommendation is based on the fact that palbociclib capsules should be taken with food to improve their absorption and reduce variability in systemic exposure, ensuring consistent therapeutic efficacy and safety [8,9,10,11].

Bioequivalence studies are conducted to determine whether different formulations containing the same active pharmaceutical ingredient, tested under identical experimental conditions, exhibit comparable bioavailability. This involves analyzing the pharmacokinetic profiles of both the test formulation (generics candidate) and the reference product and performing a bioequivalence assessment to confirm that they share the same rate and extent of absorption. In this context, the objective of the present study was to assess the bioequivalence and tolerability of two palbociclib capsule formulations to fulfill the regulatory requirements for branded generic product registration in Latin American markets.

## 2. Materials and Methods

### 2.1. Drug Products

The test formulation was a palbociclib 125 mg capsule (Palbocil^®^ or Bapocil^®^, lot number: L643C, expiry date: July 2025), manufactured by Laboratório LKM S.A. (Buenos Aires, Argentina). The reference product was an Ibrance^®^ (palbociclib) 125 mg capsule (lot number: LE6683, expiry date: January 2028), manufactured by Pfizer Manufacturing, Deutschland GmbH (Freiburg, Germany). Drug administration was performed in two periods: 20 October 2024 (Period 1) and 3 November 2024 (Period 2). Both administrations occurred within the labeled shelf-life of the test and reference products, which were stored under recommended conditions throughout the study.

The test and reference formulations have a high degree of similarity, as both formulations employ commonly used diluents, disintegrants, and lubricants. The test and reference formulations contain microcrystalline cellulose as the primary filler, lactose monohydrate as a secondary diluent, and sodium starch glycolate as a disintegrant. Additionally, both formulations include magnesium stearate as a lubricant and colloidal silicon dioxide as a glidant to improve powder flow and capsule uniformity. Minor differences are observed in the colorants and capsule shell components, which are not expected to influence drug release or absorption. Overall, the excipient profiles of the two formulations are qualitatively comparable.

### 2.2. Exploratory In Vitro Assessment

Prior to the bioequivalence study, the exploratory dissolution profiles of the test and reference formulations of palbociclib capsules were evaluated using the FDA-recommended dissolution method [12], under the following conditions:

- Apparatus: USP II (paddle);
- Rotation speed: 50 rpm;
- Medium: 0.1 N HCl;
- Volume: 900 mL (maintained at 37.0 ± 0.5 °C);
- Sampling times: 10, 15, 20, 30 and 45 min.

To further explore the conditions for comparing the formulations in vitro, dissolution profiles were also obtained in alternative media: HCl pH 1.2, acetate buffer pH 4.5, phosphate buffer pH 6.8, and purified water. As an exploratory approach, all dissolution assays were performed using six vessels for each formulation.

Samples were withdrawn at predetermined time points, filtered, and analyzed using a validated HPLC method with UV detection. The amount of palbociclib dissolved was quantified by comparing the peak areas against the calibration curves prepared for each respective medium.

The similarity between the dissolution profiles of the test and reference formulations was assessed using the f_2_ similarity factor.

### 2.3. Bioequivalence Study

#### 2.3.1. Ethical Aspects and Good Clinical Practices

The study protocol was approved by the Research Ethics Committee of the São Francisco University (Bragança Paulista, Brazil). All phases of the bioequivalence study were conducted at the Unidade Integrada de Farmacologia e Gastroenterologia—UNIFAG (Bragança Paulista, Brazil), a clinical research center certified and audited by ANVISA for bioequivalence/bioavailability studies. This study adhered to the ethical principles outlined in the Good Clinical Practices Guidelines of the Declaration of Helsinki [13], local Brazilian laws [14,15], and the requirements for bioequivalence studies [16,17,18,19]. All participants provided written informed consent before the initiation of any study procedures.

#### 2.3.2. Study Population

A total of 52 healthy Brazilian subjects (26 males and 26 females) were enrolled in the study, aged between 18 and 55 years, with a body mass index (BMI) ranging from 18.5 to 30.0 kg/m^2^. Eligibility criteria required participants to be in good general health, with no clinically significant medical conditions, as assessed by the investigator through clinical history, physical examination, vital signs, electrocardiogram, anthropometric measurements, and standard laboratory tests. Additionally, participants were required to fully understand the objectives, nature, potential risks, and possible adverse events associated with the study, as well as to provide written informed consent indicating their willingness and ability to comply with all study procedures.

In accordance with the ethical principle of non-maleficence, prophylactic administration of an antiemetic agent was employed to mitigate the incidence of nausea, a very common adverse event associated with the investigational drug (≥10%, as indicated in the prescribing information of the reference product). The antiemetic prophylaxis, Dramin^®^ B6 DL (dimenhydrinate, pyridoxine hydrochloride, glucose, and fructose), was administered at 3 and 7 h after drug administration in both periods, using the same dosage regimen (30 mg diluted in 100 mL of normal saline, infused over 30 min). No scientific evidence of pharmacokinetic interaction between palbociclib and Dramin^®^ B6 DL was identified. Furthermore, considering the different routes of administration (oral and i.v.), no interference with drug absorption occurred.

Participants were instructed to completely abstain from consuming alcoholic drinks and any food or beverage containing caffeine or xanthines, including coffee, tea, chocolate, and soft drinks made with cola or guarana, for the entire duration of the study.

#### 2.3.3. Study Design

This open-label, randomized, two-period, two-sequence crossover study was initially designed to include 52 healthy subjects (26 females and 26 males) and was completed with 50 subjects (25 females and 25 males), aged 19 to 53 years. This study was conducted with a 14-day washout, and in the fed-state.

The fed condition used a standardized high-fat, high-calorie meal (approximately 1000 kcal with ≥50% of calories from fat), consumed after an overnight fast of at least 8 h; the meal was eaten within 30 min, and the investigational products were administered 30 min after the start of the meal. All subjects received the same diet and portions in both study periods. The subjects received a single dose of 125 mg palbociclib capsule from one of the two formulations in each period, according to the randomization schedule, along with 200 mL of water. Subsequently, the study participants remained fasted for an additional four hours. Additional water intake was permitted two hours after drug administration.

As the elimination half-life of palbociclib is greater than 24 h, the collection schedule was truncated to 72 h, and a total of 22 blood samples (7.5 mL each) were collected in tubes containing K_3_EDTA (anticoagulant) at the following time points: pre-dose and post-dose at 1.00, 2.00, 3.00, 4.00, 5.00, 5.50, 6.00, 6.50, 7.00, 7.50, 8.00, 8.30, 9.00, 9.50, 10.0, 11.0, 12.0, 14.0, 24.0, 48.0, and 72.0 h. Immediately after collection, blood samples were centrifuged at 3000 rpm (1500× *g*) for 10 min at 22 °C. The separated plasma was carefully aliquoted into amber cryogenic tubes, properly labeled, and stored at −70 °C until bioanalysis.

The primary endpoint of this study was to assess bioequivalence between the test and reference formulations based on pharmacokinetic parameters C_max_ (maximum plasma concentration) and AUC_0–72_ (area under the plasma concentration–time curve from 0 to 72 h). The secondary endpoint included evaluation and descriptive analysis of t_max_ (time to reach C_max_) for both test and reference formulations. Safety and tolerability were also assessed through the monitoring and reporting of adverse events.

#### 2.3.4. Bioanalytical Method

Palbociclib was extracted from a 300 µL aliquot of human plasma via liquid–liquid extraction (1.25 mL of ethyl acetate) and using palbociclib-d8 (deuterium-labeled version of the drug) as the internal standard (IS). The samples were analyzed using high-performance liquid chromatography coupled with tandem mass spectrometry (LC-MS/MS). The system includes a Shimadzu^®^ LC-20AD (Shimadzu Corporation, Kyoto, Japan) and a Xevo^®^ TQD MS/MS (Waters Corporation, Milford, MA, USA).

An aliquot of each sample (5 µL) was injected onto an Agilent^®^ Polaris^®^ C18-A column (2.0 × 50 mm; 5 µm particle size) (Agilent Technologies Inc., Santa Clara, CA, USA), maintained at 22 °C. The mobile phase consisted of a mixture of acetonitrile, 5 mM ammonium acetate, and formic acid (600:400:1, *v*/*v*/*v*), adjusted to pH 4.0.

The detection of palbociclib was carried out in the mass spectrometer with the positive electrospray ionization multiple-reaction monitoring mode set to transmit at *m*/*z* 448.58 → 380.33 for palbociclib and *m*/*z* 456.30 → 388.54 for palbociclib-d8 (IS).

The validated bioanalytical method covered all required tests, including selectivity, analyte interference with concomitant medications, matrix effect, carryover, calibration curve, precision, accuracy, reinjection reproducibility, and stability assessments. Linearity was demonstrated across a concentration range of 1 to 250 ng/mL. To avoid inter-assay variations, all the samples from the same participant were assessed in the same analytical run. Incurred sample reanalysis (ISR) have been executed in order to verify the reliability of the reported sample analyte concentrations.

All evaluated parameters were established and conducted in accordance with the applicable guidances for bioanalytical method validation issued by INVIMA [18], ISP [19] and ANVISA [20].

#### 2.3.5. Safety

Participant safety was assessed throughout the study. Laboratory evaluations included hematology, urine analysis, and blood chemistry, along with physical examinations and electrocardiograms performed at the beginning and end of the study. Pre-study screening also included hepatitis B and C and HIV serology.

During each study period, participants were monitored for body temperature, blood pressure, and heart rate immediately before drug administration and at 3, 6, 9, and 14 h post-dose. Adverse events were monitored continuously throughout the study.

#### 2.3.6. Statistical Analysis

The sample size was calculated considering the following parameters: a significance level of 5%, a bioequivalence acceptance range of 80–125%, a statistical power of 80%, and an intra-subject CV of 22%, based on the CRO’s experience with this drug. Based on these assumptions, the estimated sample size was 52 subjects, including an allowance for potential dropouts to ensure the robustness of the statistical analysis.

The pharmacokinetic parameters were obtained from the palbociclib plasma concentration–time curves. These parameters were statistically assessed for bioequivalence analysis using Phoenix WinNonLin (version 6.4). The area under the plasma concentration–time curve was calculated using the linear trapezoidal method, from 0 to 72 h (AUC_0–72_). As the elimination half-life of palbociclib is greater than 24 h, the collection schedule was truncated to 72 h. The peak of the maximum plasma concentration (C_max_) of palbociclib and the time to reach this peak (t_max_) were obtained directly, with no data interpolation.

For the bioequivalence analysis, C_max_ and AUC_0–72_ were considered as primary endpoints. The statistical model incorporated fixed effects for sequence, period, and treatment using ANOVA, while subjects nested within sequence were considered random effects. A 90% confidence interval was calculated for the difference in the means of the ln-transformed values between the test and reference products. The antilog of this interval provided the 90% CI for the geometric mean ratio of the primary pharmacokinetic parameters. Bioequivalence was concluded when the 90% CI limits for these ratios fell within the acceptance range of 80.00% to 125.00%, as established by INVIMA (Colombia), ISP (Chile), ANVISA (Brazil), FDA (U.S.), and other regulatory agencies [10,15,18,19].

## 3. Results

### 3.1. Exploratory Dissolution Profiles

**Figure 1:**
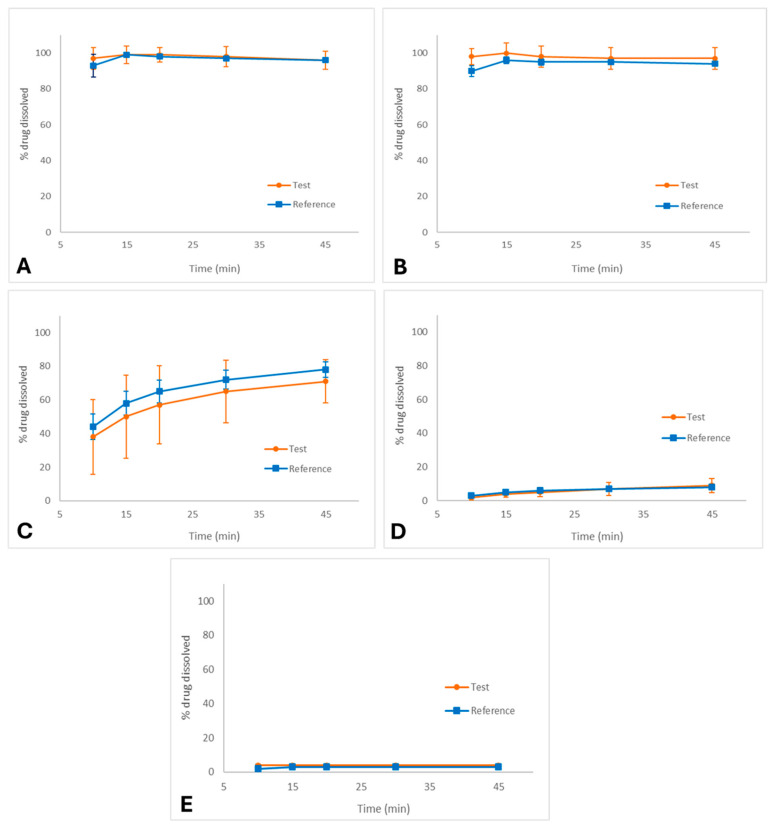
Dissolution profiles (media ± SD) of palbociclib capsules (test and reference formulations) in different media: (**A**) 0.1 N HCl (FDA method), (**B**) pH 1.2, (**C**) pH 4.5 acetate buffer, (**D**) pH 6.8 phosphate buffer, and (**E**) purified water. Conditions: USP Apparatus II (paddle), 50 rpm, 900 mL, 37.0 ± 0.5 °C.

The exploratory dissolution profiles of the test and reference palbociclib capsules in different media are shown in Figure 1A–E.

In 0.1 N HCl (FDA method, Figure 1A), both formulations exhibited rapid and complete dissolution, reaching more than 85% within 15 min, with nearly superimposable profiles. Similarly, in pH 1.2 medium (Figure 1B), dissolution was fast and complete for both products, confirming that palbociclib is highly soluble under strongly acidic environments. Under these conditions, f_2_ calculation was not required as the profiles meet the criterion for immediate release similarity (dissolution ≥ 85% at 15 min).

In pH 4.5 acetate buffer (Figure 1C), dissolution was slower and incomplete compared to acidic media, with approximately 75% dissolved at 45 min. Due to the reduced solubility of palbociclib in this environment, the dissolution profiles for both products were highly variable.

In pH 6.8 phosphate buffer (Figure 1D), dissolution was markedly reduced, not exceeding 15% after 45 min for either formulation. Similarly, in purified water (Figure 1E), dissolution was minimal (<10%), confirming the poor solubility of palbociclib in neutral aqueous environments.

Consequently, f_2_ was not calculated for pH 4.5, pH 6.8, and purified water as none of these media achieved sufficient dissolution and the data exhibited high variability, preventing a valid similarity factor assessment. Nevertheless, a visual inspection of the profiles was performed to compare the formulations under these conditions.

### 3.2. Bioequivalence

#### 3.2.1. Study Subjects

Following the evaluation of medical history, vital signs, physical examination, electrocardiogram, and standard laboratory analyses, all participants were confirmed to be in good health and free from clinically significant conditions. The demographic characteristics of the subjects are presented in Table 1.

As per protocol, a total of 52 healthy subjects (26 males and 26 females) were successfully enrolled in the study. Following discharge after the first period, one subject was withdrawn due to personal reasons. After drug administration in period 2, another subject was excluded from the study due to an adverse event (nausea and vomiting).

#### 3.2.2. Bioanalysis

The method validation encompassed every necessary parameter, including selectivity, interference from co-administered medications, matrix effect, carry-over, calibration curve, precision, accuracy, reinjection reproducibility, and stability evaluations.

The method exhibited linearity across the concentration range of 1.0 to 250.0 ng/mL, with a lower limit of quantification (LLOQ) of 1.0 ng/mL. Selectivity was confirmed by demonstrating that endogenous compounds present in blank plasma did not interfere with the retention times of palbociclib or the IS (palbociclib-d8), including lipemic and hemolyzed plasma samples. The responses of interfering peaks close to analyte retention time were lower than 20% of the analyte response in LLOQ samples, and the responses of interfering peaks close to IS retention time were lower than 5% of the IS response, where the same was considered for the carry-over effect. For the matrix effect, the coefficient of variation (CV) of internal standard normalized matrix factor related to all samples was lower than 15%.

For the approval of calibration standards, deviation was smaller than or equal to 20% compared to the nominal concentration for LLOQ patterns, and it was smaller than or equal to 15% compared to other calibration standards. The acceptance criteria for the calibration curves were to have a minimum of 75% of the calibration standards approved according to the previous criteria and at least 6 calibration standards of different concentrations, including LLOQ and upper limit of quantification (ULOQ).

Regarding precision and accuracy (intra and inter-run), coefficient of variation values above 15% were not permitted, except for LLOQ, for which values less than or equal to 20% were accepted.

Stability studies confirmed that plasma samples remained within 15% of the nominal concentration for up to 30 h at room temperature (15–25 °C) and were stable for as long as 72 h post-extraction when stored in the auto-sampler at 22 °C. Freeze–thaw evaluations showed that samples retained stability after three cycles of freezing at −20 °C and −70 °C, followed by thawing at room temperature. Additionally, long-term stability was demonstrated, with samples remaining stable for up to 214 days when kept in an ultrafreezer at −70 °C. These findings ensure appropriate storage conditions prior to analysis, supporting accurate drug quantification.

The absence of palbociclib in all pre-dose plasma samples demonstrated that no carryover occurred and that the washout period was properly maintained.

ISR assay was adequate, showing that the percentage difference in the results between the original data and the repeat study was ≤20% in 97.5% of the reanalyzed samples. ISR is an important component of bioanalytical method application and verifies the reliability of the reported analyte concentrations [19].

All evaluated parameters met the acceptance limits specified by applicable bioanalytical method validation guidances [18,19,20].

#### 3.2.3. PK Analysis

A total of 52 healthy volunteers were enrolled in the study, and 50 participants (25 females and 25 males) successfully completed both study periods, qualifying for inclusion in the pharmacokinetic and statistical analyses.

**Figure 2:**
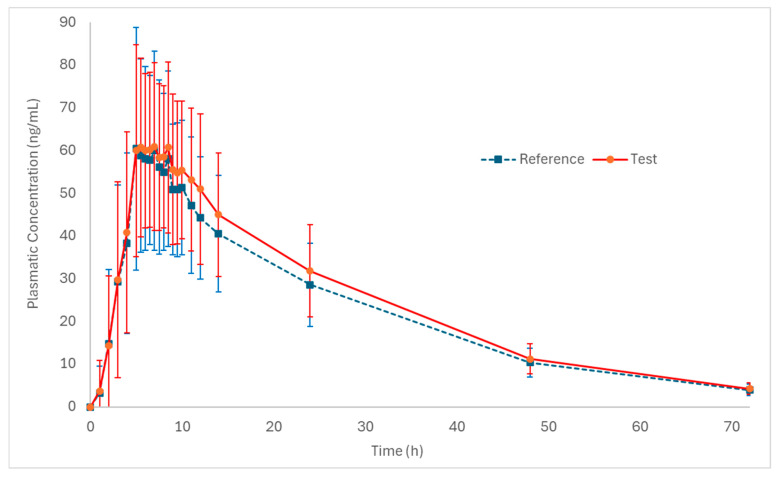
Pharmacokinetic profiles (plasmatic concentration vs. time) of the test and reference formulations in healthy subjects (n = 50) under fed conditions (mean ± SD).

**Figure 3:**
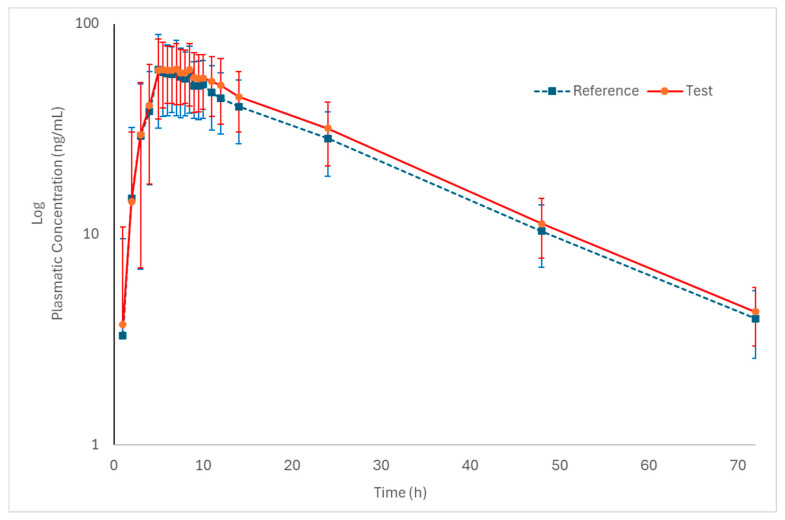
Log-transformed pharmacokinetic profiles of the test and reference formulations in healthy subjects (n = 50) under fed conditions (mean ± SD).

Figure 2 and Figure 3 (log-transformed) show the mean plasma concentration vs. time curves for palbociclib when administered under fed conditions. Both test and reference formulations exhibited similar pharmacokinetic behavior. In addition, sampling intervals were sufficient to correctly capture the absorption phase and the elimination phase of the drug.

Table 2 presents the primary pharmacokinetic parameters of palbociclib for both the test and reference formulations.

**Figure 4:**
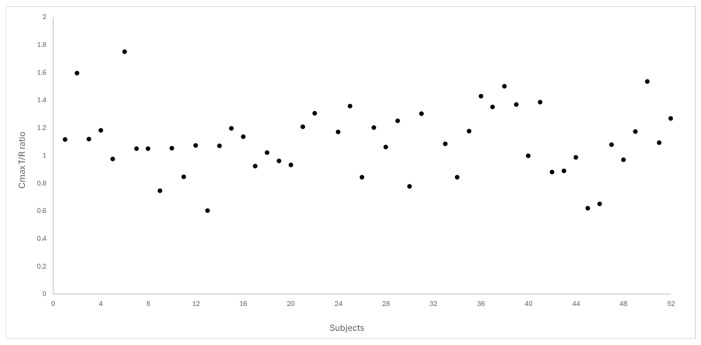
Dispersion of T/R ratio for C_max_ between the study subjects (N = 50).

**Figure 5:**
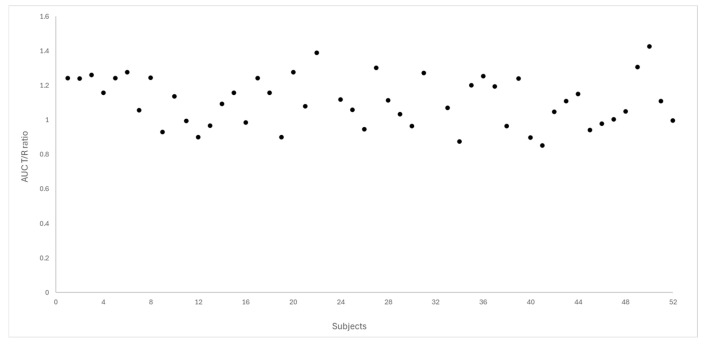
Dispersion of T/R ratio for AUC_0–72_ between the study subjects (N = 50).

Figure 4 and Figure 5 depict the distribution of T/R ratios for C_max_ and AUC_0–72_ among the 50 subjects who completed both study periods. The data demonstrate a narrow dispersion, indicating low intra-subject variability between formulations for these pharmacokinetic metrics. This observation is quantitatively supported by the within-subject coefficient of variation (CV_ws_), which was calculated as 14.6% for C_max_ and 9.0% for AUC_0–72_, as summarized in Table 3.

#### 3.2.4. Bioequivalence Outcome

Table 3 presents the T/R geometric mean ratio and the 90% CIs for pharmacokinetic parameters C_max_ and AUC_0–72_ (truncated) obtained from the bioequivalence analysis.

**Table 3:** Geometric mean ratio and 90% confidence intervals from the bioequivalence analysis.

Parameter *	N	T/R Geometric Mean Ratio %	90% Confidence Interval	CV_ws_ %	Statistical Power %
C_max_	50	107.07	101.98–112.42	14.6	>99.9
AUC_0–72_ (truncated)	50	109.77	106.51–113.13	9.0	>99.9

* Ln-transformed; CV_ws_, within-subject coefficient of variation.

As the 90% confidence intervals for the test/reference geometric mean ratios of all pharmacokinetic measures complied with the acceptance criteria of 80.00–125.00%, both formulations (test and reference) were considered bioequivalent in terms of the rate and extent of absorption.

#### 3.2.5. Safety

Thirty non-serious adverse events were reported in 23 of 52 subjects: sixteen in the test formulation, eleven in the reference product, and three occurred before drug administration. All adverse events were considered to be of mild intensity. The most common non-serious adverse events were urine analysis abnormal (17%), nausea, headache, and white blood cell analysis abnormal (10% each), as described in Table 4. Regarding causality, among the most frequent adverse events, only nausea and ALT increased were classified as possibly related to drug administration.

Regarding sex, the distribution of reported adverse events was well balanced, with 16% occurring in females and 14% in males. In conclusion, both test and reference products were well tolerated, with no serious adverse events observed and no clinically significant findings related to tolerability.

## 4. Discussion

### 4.1. Exploratory In Vitro Assays

The exploratory dissolution profiles confirmed that palbociclib exhibits pronounced pH-dependent solubility, which is consistent with its weakly basic nature. Rapid and complete dissolution was observed under strongly acidic conditions (0.1 N HCl and pH 1.2), supporting its classification as highly soluble in low pH environments. Conversely, dissolution decreased substantially in media with higher pH values, such as acetate buffer (pH 4.5), phosphate buffer (pH 6.8), and purified water, where incomplete and highly variable profiles were obtained. Despite the adverse conditions in some media, the exploratory dissolution profiles were valuable for comparing the test and reference products prior to the bioequivalence study, revealing that these pH conditions may be useful to support the development of palbociclib formulations.

### 4.2. Bioequivalence and Tolerability Assessment

For the regulatory approval of generic palbociclib capsule formulations, both the FDA and the EMA recommend conducting a single-dose bioequivalence study in healthy volunteers under fed conditions [10,11]. The present in vivo study demonstrated the bioequivalence of test and reference palbociclib 125 mg capsules, and it also monitored safety and tolerability of the formulations when administered under fed conditions in healthy subjects. The pharmacokinetic parameters obtained for the test product were comparable to those observed for the reference product. The 90% confidence intervals of T/R geometric mean ratios were 101.98–112.42% for C_max_ and 106.51–113.13% for AUC_0–72_, which are all within the accepted bioequivalence range of 80.00–125.00%.

The values obtained for the pharmacokinetic parameters of the test (C_max_: 74.7 ng/mL; AUC_0–72_: 1703.9 ng·h/mL and t_max_: 6.5 h) and reference (C_max_: 71.4 ng/mL; AUC_0–72_: 1564.7 ng·h/mL and t_max_: 6.5 h) formulations in the present study are consistent with the pharmacokinetic data found in the literature, further supporting the reliability of the results and the adequacy of the study design for assessing bioequivalence.

In the study conducted by Hu et al. (2025) [21], a two-period, two-sequence replicated crossover design was used to evaluate the bioequivalence of palbociclib 125 mg capsules under fasting and fed conditions. In the fed arm, the reference product (Ibrance^®^) showed a mean C_max_ of 84.6 ng/mL, an AUC_0–t_ of 2394 ng·h/mL, and a t_max_ of 4.99 h. The authors concluded that food intake did not significantly affect the absorption of palbociclib, supporting its consistent pharmacokinetic profile across dietary conditions.

Arumugam et al. (2023) [22] conducted a bioequivalence study (semi-replicate crossover design) of palbociclib 125 mg capsules under fed conditions in 48 healthy Indian male subjects, of whom 44 completed the study. The pharmacokinetic analysis revealed that the test formulation had a mean C_max_ of 67.6 ± 15.5 ng/mL, AUC_0–72_ of 1894 ± 364.7 ng·h/mL, and median t_max_ of 6.75 h (4.50–12.0 h), while the reference formulation (Ibrance^®^) showed a mean C_max_ of 60.1 ± 14.1 ng/mL, AUC_0–72_ of 1766 ± 297.8 ng·h/mL, and median t_max_ of 8.00 h (4.50–24 h).

Chu et al. (2021) [23] conducted two independent bioequivalence studies (fasted and fed) of the 125 mg palbociclib capsule in healthy Chinese subjects. These studies were open-label, randomized, two-period crossover, and single-dose, and samples were collected up to 120 h post-dose. A total of 30 subjects were included in each study, with all 30 subjects finishing the fasting study and 29 completing the fed study. In the fed study, the values for the pharmacokinetic parameters of the reference drug were 57.6 ng/mL for C_max_, 1608.10 ng·h/mL for AUC_0–t_, and 7.00 h for t_max_.

In the study by Ruiz-García et al. (2017) [24], the pharmacokinetics of the 125 mg palbociclib capsules were evaluated under four dietary conditions: high-fat, low-fat, moderate-fat, and fasting. Across all fed treatments, the mean C_max_ ranged from 48.64 to 53.67 ng/mL, and the AUC_0–inf_ ranged from 1573 to 1672 ng·h/mL. The median t_max_ was consistent at 8.00 h for all conditions. These results indicated no significant differences in the absorption rate of palbociclib across the different dietary regimens.

Regarding safety, the formulations administered in our study were well-tolerated, with no serious adverse events reported. The non-serious events reported with a prevalence of ≥10% were urine analysis abnormal (17%), nausea, headache, and white blood cell analysis abnormal (10% each). All adverse events were considered to be of mild intensity. It is important to exercise caution when evaluating adverse events resulting from a bioequivalence study; this study was conducted with healthy subjects in a controlled trial, and the drug formulations were administered as a single dose.

Finally, to the best of our knowledge, this is the first published bioequivalence study of the palbociclib capsule conducted in a Latin American population.

## 5. Conclusions

This study successfully characterized the pharmacokinetic profile of palbociclib for both the test and reference formulations. Both products were well tolerated and exhibited comparable safety profiles in healthy volunteers under fed conditions. The 90% confidence intervals for the geometric mean ratios (test/reference) of log-transformed C_max_ and AUC_0–72_ values fell within the predefined bioequivalence limits of 80.00–125.00%. It was demonstrated that the test product satisfies the bioequivalence criteria regarding the reference formulation in terms of its absorption characteristics, supporting the expectation of comparable therapeutic outcomes.

## Figures and Tables

**Table 1:** Demographic characteristics of the study subjects (N = 50).

Characteristic	Descriptive Statistics
Age (years)	
Mean _(±SD)_	32.8 _(±9.05)_
Range	19–53
Weight (kg)	
Mean _(±SD)_	72 _(±11)_
Range	50–95
Height (m)	
Mean _(±SD)_	1.66 _(±0.08)_
Range	1.50–1.85
BMI (kg/m^2^)	
Mean _(±SD)_	25.94 _(±2.57)_
Range	19.5–29.8
Gender	
Male	25 _(50%)_
Female	25 _(50%)_

**Table 2:** Pharmacokinetic parameters of the palbociclib capsule formulations (test and reference) in healthy subjects under fed conditions (n = 50, male and females).

Parameter	Test	Reference
t_max_ (h) Median (min–max)	6.5 (5.0–12)	6.5 (5.0–10)
C_max_ (ng/mL) Mean ± SD	74.7 ± 21.5	71.4 ± 28.4
AUC_0–72_ (h·ng/mL) Mean ± SD	1703.9 ± 467.7	1564.7 ± 493.0

C_max_: maximum plasma concentration; t_max_: time to reach the maximum plasma concentration; AUC_0–72_: area under the curve of plasma concentration vs. time from 0 to 72 h.

**Table 4:** Non-serious adverse events reported during the bioequivalence study (total of 30 events reported) by 23 of the 52 study subjects.

Adverse Event ^1^	% ^2^	Causality ^3^	Intensity
Urine analysis abnormal	17	Unlikely	Mild
Nausea	10	Possible	Mild
Headache	10	Unlikely	Mild
WBC analysis abnormal	10	Unlikely	Mild
ALT increased	6.7	Possible	Mild
Blood pressure increased	6.7	Unrelated	Mild
Others	≤3.3		

^1^ Terminology based on MedDRA. ^2^ Calculated based on the 30 adverse events reported during the study. ^3^ Based on WHO-UMC causality categories. WBC, white blood cell. ALT, alanine aminotransferase.

## Data Availability

The original contributions presented in this study are included in the article. Further inquiries can be directed to the corresponding author.
